# A Novel Approach for Modeling Neural Responses to Joint Perturbations Using the NARMAX Method and a Hierarchical Neural Network

**DOI:** 10.3389/fncom.2018.00096

**Published:** 2018-12-06

**Authors:** Runfeng Tian, Yuan Yang, Frans C. T. van der Helm, Julius P. A. Dewald

**Affiliations:** ^1^Department of Physical Therapy and Human Movement Sciences, Feinberg School of Medicine, Northwestern University, Chicago, IL, United States; ^2^Department of Biomechanical Engineering, Northwestern University, Evanston, IL, United States; ^3^Department of Biomechanical Engineering, Delft University of Technology, Delft, Netherlands

**Keywords:** neural modeling, NARMAX, neural network, EEG, non-linear system identification

## Abstract

The human nervous system is an ensemble of connected neuronal networks. Modeling and system identification of the human nervous system helps us understand how the brain processes sensory input and controls responses at the systems level. This study aims to propose an advanced approach based on a hierarchical neural network and non-linear system identification method to model neural activity in the nervous system in response to an external somatosensory input. The proposed approach incorporates basic concepts of Non-linear AutoRegressive Moving Average Model with eXogenous input (NARMAX) and neural network to acknowledge non-linear closed-loop neural interactions. Different from the commonly used polynomial NARMAX method, the proposed approach replaced the polynomial non-linear terms with a hierarchical neural network. The hierarchical neural network is built based on known neuroanatomical connections and corresponding transmission delays in neural pathways. The proposed method is applied to an experimental dataset, where cortical activities from ten young able-bodied individuals are extracted from electroencephalographic signals while applying mechanical perturbations to their wrist joint. The results yielded by the proposed method were compared with those obtained by the polynomial NARMAX and Volterra methods, evaluated by the variance accounted for (VAF). Both the proposed and polynomial NARMAX methods yielded much better modeling results than the Volterra model. Furthermore, the proposed method modeled cortical responded with a mean VAF of 69.35% for a three-step ahead prediction, which is significantly better than the VAF from a polynomial NARMAX model (mean VAF 47.09%). This study provides a novel approach for precise modeling of cortical responses to sensory input. The results indicate that the incorporation of knowledge of neuroanatomical connections in building a realistic model greatly improves the performance of system identification of the human nervous system.

## Introduction

The human nervous system is an integrated, large-scale system consisting of connected neuronal networks. The coordination of neural activity across networks, between the periphery and the central nervous system, is essential for fulfilling our daily functions including movement control and sensory perception. Modeling and system identification of the human nervous system helps us understand how the brain processes sensory input and controls behavior. Efforts have been made through previous studies on building a mathematical model of the human nervous system from neurons and circuits to large-scale neural networks. As yet, challenges remain, because of substantial non-linearity and fast dynamics in the nervous system (Breakspear, 2017). The non-linearity of the nervous system enables the rich, task-relevant neural encoding, and communication, while the fast dynamics (including both linear and non-linear components) allow for efficient processing and transmission of neural information (Friston, 2000). However, these two basic properties of the human nervous system, as well as the poor signal-to-noise of measured neural signals, significantly increase the difficulty of building a precise model to describe the behavior of the human nervous system.

A typical way to investigate the input-output behavior of the human nervous system is to apply an external peripheral input and to measure the neural response from brain. Electroencephalography (EEG) is a non-invasive electro-neurophysiological technique widely applied to measure neural responses from the brain to external inputs. When providing periodic stimulation with specific frequencies, a linear system only generates phase-locked responses at the stimulated frequencies while a non-linear system can produce cross-frequency phase-coupled responses at non-stimulated frequencies (Langdon et al., 2011; Billings, 2013; Yang et al., 2016b). When applying angular position perturbations to the wrist joint, a recent study demonstrated that more than 80% signal power of brain activity resulting from the movement stimulation occurs in the non-stimulated frequencies and only 10% of brain activity could be explained using the best linear approximation model (Vlaar et al., 2017). As a result, a non-linear approach is necessary for modeling the brain response to wrist joint perturbations. Furthermore, previous EEG studies have reported sub-harmonic responses (i.e., the response frequency is a fraction of a stimulus frequency) in the brain activity following joint perturbations, indicating complicated non-linear dynamics of cortical oscillations (Yang et al., 2016a; Breakspear, 2017). Such an ill-posed non-linear phenomenon cannot be explained by classical non-linear models such as a Wiener and Hammerstein system configuration (i.e., a series connection of static non-linear blocks with a dynamic linear block; Crama and Schoukens, 2001; Paduart et al., 2012), though they have been previously used for system identification and modeling of the periphery system including human musculoskeletal systems (Westwick and Kearney, 2001; Dempsey and Westwick, 2004).

The human nervous system comprised of multiple neuronal circuits resulting in a complicated closed-loop non-linear system with fast dynamics (in the order of millisecond). Previous studies tried to use a simplified approach based on Volterra series to model the brain response to joint perturbations. This only explained around 40% of the measured EEG signal (Vlaar et al., 2018). Volterra series is a polynomial functional expansion similar to a Taylor series that provides an approximation of input-output relation in a non-linear system (Brockett, 1976). A serious limitation of Volterra series based approaches is that it completely ignores the closed-loop behaviors of the nervous system as they contain no autoregressive terms. To address this limitation, we proposed to use the non-linear autoregressive moving average with exogenous inputs (NARMAX) model (Chen and Billings, 1989; Chen et al., 1989). The NARMAX model is a powerful tool for black-box system identification problems, in particular when limited knowledge about the detailed model structure of the system is available. A wide range of non-linear systems can be well represented using the NARMAX method, including those with exotic non-linear behaviors such as subharmonics, bifurcations, and even chaos, as previously reported for the human nervous system (Breakspear, 2017).

The classical NARMAX model is based on polynomial expansions, which makes it difficult to precisely mimic properties of the nervous system such as the sigmoid function of a synapse. The model error may cumulatively increase when the neural pathway contains more than one synapse as in the somatosensory afferent pathways from muscles to the brain. To address this challenge, we replaced the polynomial non-linear terms with a hierarchical neural network. The hierarchical neural network is built based on known neuroanatomical connections and corresponding transmission delays in neural pathways. The proposed method is evaluated using an experimental dataset, where brain responses to joint perturbations at the wrist in able-bodied individuals were obtained by EEG signals.

## Data Acquisition and Preprocessing

### Data Acquisition

The experimental data were recorded from ten young able-bodied individuals (age range 22–25 years old; 6 men; all right-handedness). The experimental procedure was approved by the Human Research Ethics Committee of the Delft University of Technology, the Netherlands. All participants signed informed consent before the experiments.

During the experiment, subjects sat next to a wrist manipulator (WM), which is an actuated rotating device with a single degree of freedom to exert flexion and extension perturbations to wrist joint (Wristalyzer, Moog Inc., The Netherlands). The lower arm (in the dominant side) of subjects was strapped in the armrest while the hand closely touched the handle of the manipulator and fixed with velcro. The perturbations were applied as flexion and extension stretches to the participants as the external input to the nervous system via the handle of the manipulator. Participants were instructed to relax their arm and fingers, and not react to the perturbations during the experiment.

The stimulation signals consisted of the sum of sinusoids with the frequencies of 1, 3, 5, 7, 9, 11, 13, 15, 19, and 23 Hz and with the period of 1 s. Seven different sum-of-sinusoidal signals (with the same frequencies) were generated based on different relative phases between sinusoids. All stimulation signals have similar statistical distributions with the same root-mean-square (i.e., the square root of the arithmetic mean of the squares of time series) that equals to 0.02 radians. The simulation signal was applied to wrist joint as angular position perturbations, therefore the unit of signal magnitude is in radians. The signals were designed to have the equal power on the first three frequency components (i.e., 1, 3, 5 Hz) and a decaying power spectrum (−20dB/decade slope) for rest frequency components. This design is a trade-off between reduced predictability of signal (to prevent the anticipation of participants during the experiment) and capabilities of the human wrist joint and the manipulator (Vlaar et al., 2017). Each signal was applied to stimulate the wrist joint for 7 trials of 36 s per trial.

EEG was recorded from the nervous system, using a 128-channel cap (5/10 systems, WaveGuard, ANT Neuro, Germany) with Ag/AgCl electrodes, using a common average reference. The EEG and movement stimulation were digitalized at 2,048 Hz using a Refa amplifier (Twente Medical Systems International B.V., the Netherlands) and stored for further analysis. Three seconds in the beginning and the end of each trial were removed from further analysis to reduce the effect of transient dynamics, resulting in 30 × 7 = 210 periods for each participant and each stimulation.

### Data Preprocessing

We used Independent component analysis (ICA) (Makeig et al., 1996) to extract the cortical source activities as the output of the nervous system for modeling purposes. Before applying ICA, the continuous EEG signals were filtered by a 1–100 Hz zero-phase shift band-pass filter to remove possible high-frequency noise from neck muscles and slow trends in the data (e.g., blood pressure, heartbeat, breathing and sweat potentials). Notch filters implemented in Fieldtrip toolbox (Oostenveld et al., 2011) were applied to remove the 50 Hz line power noise and its harmonic distortions.

ICA was performed using the Infomax algorithm (Bell and Sejnowski, 1995) to decompose filtered EEG signals into independent source components by minimizing of mutual information among the data projections or maximizing joint entropy (Raimondo et al., 2012). Subsequently, all signals were resampled to 256 Hz (*N* = 256 samples) and then were segmented into 1-s periods according to the perturbation signal. The signal-to-noise ratio (SNR) of each ICA component was calculated using the algorithm developed by Vlaar et al. (2015). The ICA component with the highest SNR was selected as the system output for each dataset for the modeling. A dipole fitting algorithm implemented in the Fieldtrip toolbox (Oostenveld et al., 2011) was used to estimate the source locations of selected ICA components. The procedure is the same as the previous modeling study using Volterra model in the same datasets (Vlaar et al., 2018).

## Modeling based on NARMAX and Hierarchical Neural Network

### NARMAX Method

The non-linear autoregressive moving average with exogenous inputs (NARMAX) method provides a generalized framework for mathematical modeling of a non-linear closed-loop system (Chen and Billings, 1989; Chen et al., 1989). The input-output relationship of a non-linear dynamic system can be generally represented using the NARMAX method as follows:

(1) $$\begin{array}{l} y(k)=f(y(k-1),\ldots ,y\left(k-n_{y}\right),u(k-d),\\ \;u(k-d-1),\ldots ,u\left(k-d-n_{u}\right),\\ \;e(k-1),\ldots ,e\left(k-n_{e}\right)) \end{array}$$

where *k* is the time sample, *y*(*k*), *u*(*k*), and *e*(*k*) are the output, input and prediction error, respectively; *n*_*y*_, *n*_*u*_, and *n*_*e*_ are the associated maximum memory lags, *d* is the shortest input-output time delay in the system and *f*(·) is a non-linear function. The NARMAX method has been previously applied to complex real-world problems in various research fields including medical (Billings et al., 2013) and neurophysiological sciences (Li et al., 2015). The classical NARMAX model uses a polynomial expansion (e.g., *u* (*k*−*d*) *u* (*k*−*d*) + *u* (*k*−*d*) *u* (*k*−*d*−1) + …+ *u* (*k*−*d*−*n*_*u*_) *u* (*k*−*d*−*n*_*u*_) + *y* (*k*−1) *u* (*k*−*d*) +…+ *y* (*k*−*n*_*y*_) *u* (*k*− *d*−*n*_*u*_) + *y* (*k*−1) *y* (*k*−1) + *y* (*k*−1) *y* (*k*−2) + …) as the non-linear function. Although most non-linear functions can be represented by this polynomial expansion, it is difficult to precisely mimic the neuronal behavior of the nervous system as the sigmoid function. Thus, we proposed to use a hierarchical neural network to replace the non-linear polynomial expansion in the NARMAX framework for modeling the neural response.

### NARMAX Framework Based Hierarchical Neural Network (NARMAX-HNN) Model

The proposed model is built based on known neuroanatomical connections and corresponding transmission delays in neural pathways for processing the somatosensory information received from the proprioceptors in the periphery during the movement stimulation (Carpenter and Sutin, 1983; Standring, 2016). Both position and velocity of the applied joint perturbations are sensed by the muscle spindles and then transmitted by primary (group Ia) and secondary (group II) afferents to the central nervous system. Thus, we used both position (the perturbation signal) and velocity (its first-order derivative) signals as the inputs in the modeling. The Ia afferent pathway transmits both position (*p*) and velocity (*v*) information, while II afferent pathway transmits only position (*v*) information (Carpenter and Sutin, 1983; Standring, 2016). The NARMAX framework, therefore, is extended as:

(2) $$\begin{array}{l} y(k)=f(y(k-1),\ldots ,y\left(k-n_{y}\right),v\left(k-d_{1}\right),\\ \;v\left(k-d_{1}-1\right),\ldots ,v\left(k-d_{1}-n_{1}\right),p\left(k-d_{1}\right),\\ \;p\left(k-d_{1}-1\right),\ldots ,p\left(k-d_{1}-n_{1}\right),p\left(k-d_{2}\right),\\ \;p\left(k-d_{2}-1\right),\ldots ,p\left(k-d_{2}-n_{2}\right),\\ \;e(k-1),\ldots ,e\left(k-n_{e}\right)) \end{array}$$

for this dual-input-single-output model, where *v*(*k*) represents the velocity signal and *p*(*k*) the position signal, *d*_1_ is the shortest delay in the group Ia afferent pathway for transmitting the velocity and position signals, and *d*_2_ is the shortest delay in the group II afferent pathway for transmitting the position signal. The parameters of *n*_1_ and *n*_2_ indicate the system memory in group Ia and II afferent pathways, respectively. The Ia afferent pathway has the fastest nerve conduction velocity with the shortest delay from the periphery (wrist muscle receptors) to the brain. Previously electric-neurophysiological studies demonstrated that the shortest transmission delay of brain response to the somatosensory stimulation is around 16–20 ms for young able-bodied individuals (Desmedt and Cheron, 1980; Buchner et al., 1995; Campfens et al., 2015). The nerve conduction velocity for group Ia afferent pathway (80–120 m/s) is around twice of group II afferent pathway (33–75 m/s) (Buchthal and Rosenfalck, 1966; Siegel and Sapru, 2006). Thus, we set the *d*_1_ = 4 (around 16 ms for sampling rate 256 Hz), *d*_2_ = 8 (around 32 ms), *n*_1_ = *n*_2_ = 2 (around 8 ms).

**Figure 1:**
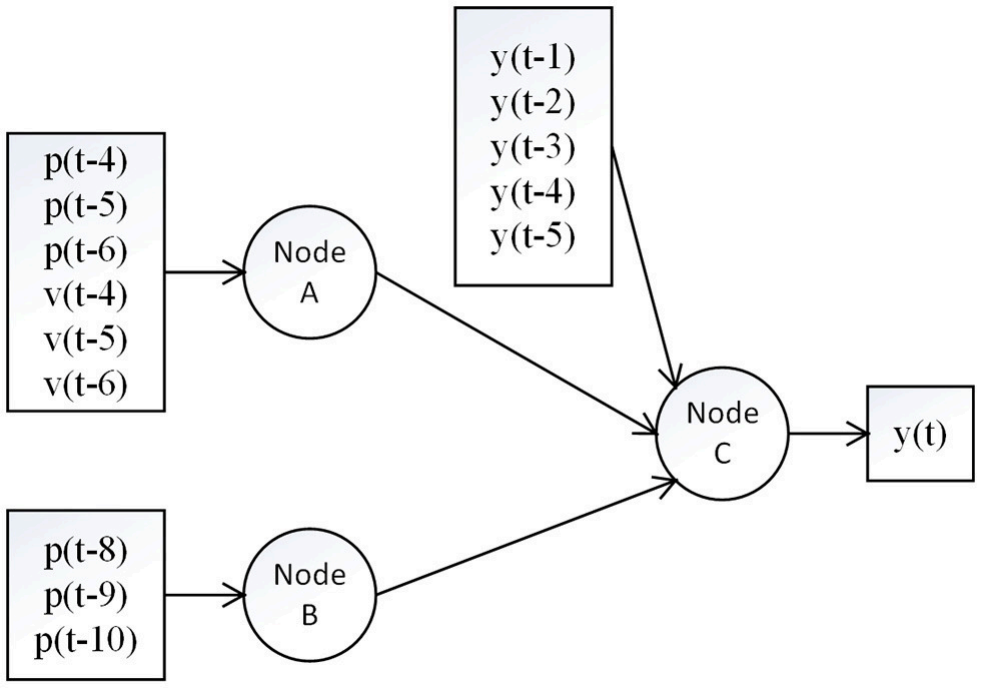
Structure of the proposed model. The signals *p, v* and *y* represent position input, velocity input and feedback interaction, respectively. Node A and B are in the first layer at the medulla, and Node C is in the second layer at the thalamus. The pathway from A to C indicates the group Ia afferent pathway which transmits both velocity and position information with a short time delay, while the pathway from B to C indicates the group II afferent pathway which transmits only the position information with a long time delay.

There are two synaptic connections in the afferent pathways from muscle spindles to the brain. The first synaptic connection is in the medulla (nucleus cuneatus) and the second one is in the thalamus (ventral posterior nucleus; Carpenter and Sutin, 1983). Thus, we built a hierarchical neural network with two layers. The first layer represents the synaptic connection in the medulla and the second layer represents the synaptic connection in the thalamus. The position and velocity information is thought to be integrated in the thalamus (Kandel et al., 2000). The structure of the proposed model is provided in Figure 1.

**Figure 2:**
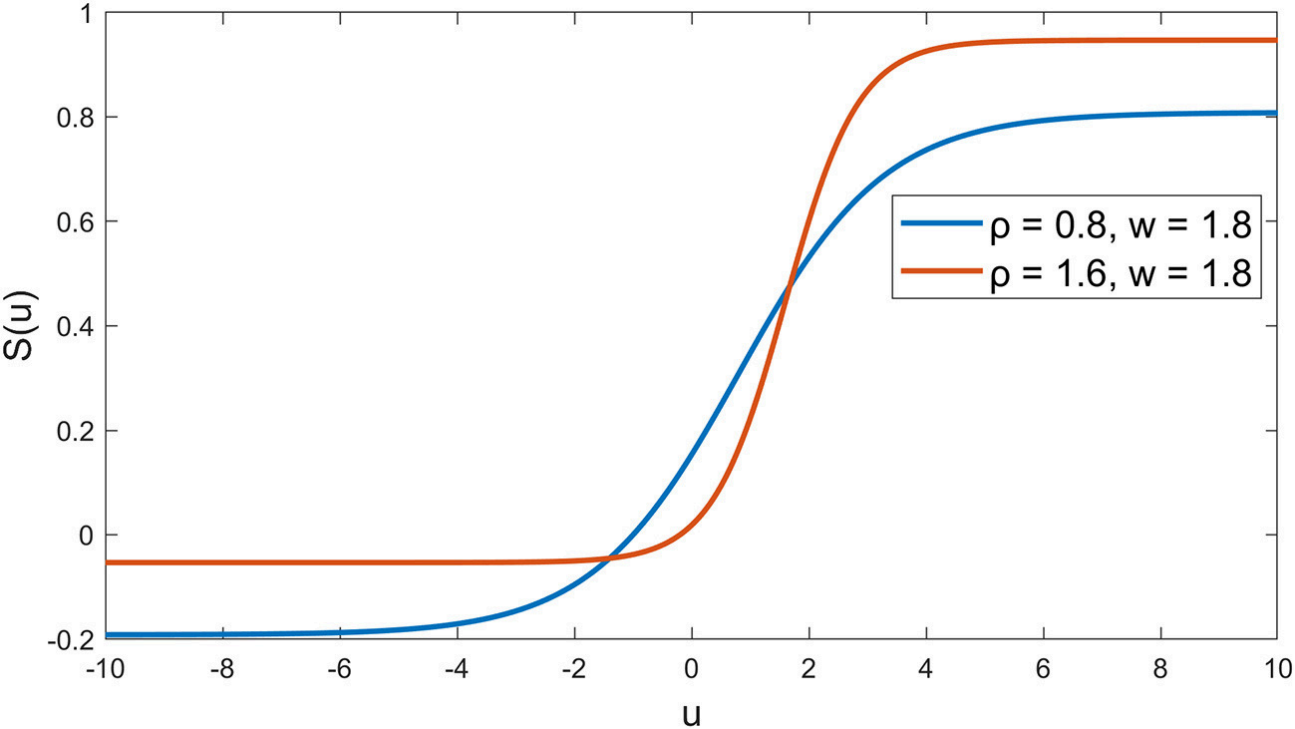
Sigmoid functions for the nodes. Blue curve indicates the synaptic behavior in the first layer at the medulla and red curve indicates the synaptic behavior in the second layer at the thalamus (Marreiros et al., 2008).

The synaptic behavior was modeled with a sigmoid function S(u) = 1/(1+exp(-ρ(u-w))-1/(1+exp(ρw))) (Moran et al., 2007) with slope parameters ρ = 0.8 and firing threshold *w* = 1.8 for nodes A and B in the first layer, and ρ = 1.6, *w* = 1.8 for the node C in the second layer (see Figure 2), which was in line with a previous neural modeling study in the human somatosensory pathway (Marreiros et al., 2008). The autoregressive terms y(k-1), …, y(k-n_y_) was added to the second layer, which represents the feedback interaction in neural circuits of the thalamocortical radiation, where the *n*_*y*_ was set to be 5 (around 20 ms). The total number of parameters in our model is 19, including 6 weights for *p* and *v* through node A, 3 weights for *p* through node B, 5 weights for *y* and 2 weights for node A and B through node C, and another 3 bias parameters for nodes A, B and C.

The model was built and trained using Neural Network Toolbox in MATLAB R2018a. The training function was selected as the Scaled Conjugate Gradient (using MATLAB function *trainscg.m*), and the performance function was selected as the Mean Squared Error (using MATLAB function *mse.m*). The signals *p, v*, and *y*, as well as output of the nodes, were normalized to values between −1 and 1 to make them in the same scale.

### Model Evaluation

Performances of the proposed model were evaluated by the Variance Accounted For (VAF) in a cross-validation test:

(3) $$VAF=\left[1-\frac{var(\hat{y}-y)}{var(y)}\right]\times 100\%$$

where *ŷ* represents the estimated output by our model and y represents the measured output from the system. For each participant, six trials were used to train the model and the rest one trial was used to test the performance of the estimated model. For each trial, there is 30 periods of 1 s data with 256 points per second (sampling rate: 256 Hz). The training and testing procedures were repeated seven times, using each trial as the testing data in the cross-validation. The mean VAF across seven repetitions was reported for each participant as the performance of the proposed model.

The obtained results were compared with those from using the polynomial NARMAX (Chen and Billings, 1989) and Volterra (Vlaar et al., 2018) models. In the Volterra model, the output depends *only* on the history of the input to the non-linear system:

(4) $$\begin{array}{l} y(k)=f(u(k-d),\;u(k-d-1),\ldots ,u\left(k-d-n_{u}\right),\\ \;e(k-1),\ldots ,e\left(k-n_{e}\right)) \end{array}$$

Thus, the Volterra model can be considered as a special case of the NARMAX model, which does not contain any autoregressive (e.g., y(k-1)y(k-1)) or interaction (e.g., y(k-1)u(k-d)) model terms.

For the NARMAX models (both the proposed model and the polynomial NARMAX model), we compared the estimated outputs from one-step-ahead (OSA) and multi-steps-ahead (MSA) predictions. The step-ahead prediction is defined as below:

(i) one-step-ahead model predicted output:

(5) $$\begin{array}{l} \hat{y}(k)=f(y(k-1),\ldots ,y\left(k-n_{y}\right)\text{,}\\ \;v\left(k-d_{1}\right),\ldots ,v\left(k-d_{1}-n_{1}\right),p\left(k-d_{1}\right),\ldots ,\\ \;p\left(k-d_{1}-n_{1}\right),p\left(k-d_{2}\right),\ldots ,p\left(k-d_{2}-n_{2}\right)) \end{array}$$

(ii) two-step-ahead model predicted output:

(6) $$\begin{array}{l} \hat{y}(k+1)=f(\hat{y}(k),y(k-1),\ldots ,y\left(k-n_{y}\right),\\ \;v\left(k+1-d_{1}\right),\ldots ,v\left(k+1-d_{1}-n_{1}\right),\\ \;p\left(k+1-d_{1}\right),\ldots ,p\left(k+1-d_{1}-n_{1}\right),\\ \;p\left(k+1-d_{2}\right),\ldots ,p\left(k+1-d_{2}-n_{2}\right)) \end{array}$$

(iii) three-step-ahead model predicted output:

(7) $$\begin{array}{l} \hat{y}(k+2)=f(\hat{y}(k+1),\hat{y}(k),\\ \;y(k-1),\ldots ,y\left(k-n_{y}\right),v\left(k+2-d_{1}\right),\ldots ,\\ \;v\left(k+2-d_{1}-n_{1}\right),p\left(k+2-d_{1}\right),\ldots ,\\ \;p\left(k+2-d_{1}-n_{1}\right),p\left(k+2-d_{2}\right),\ldots ,\\ \;p\left(k+2-d_{2}-n_{2}\right)) \end{array}$$

In this study, we evaluated the NARMAX model up to the three-step ahead prediction. Considering that dynamics of brain activity is typically in the order of few milliseconds, the three-step ahead prediction estimated 12 ms ahead EEG oscillations (based on the 256 Hz sampling rate), which is sufficient for testing the predictive performance of models. Noteworthy, for the Volterra model, there are no autoregressive variables. Consequently, it is not possible to generate the multi-step ahead prediction results for the Volterra model. The statistical significance of results is indicated by the paired *t*-test with *p* < 0.05.

## Results

**Figure 3:**
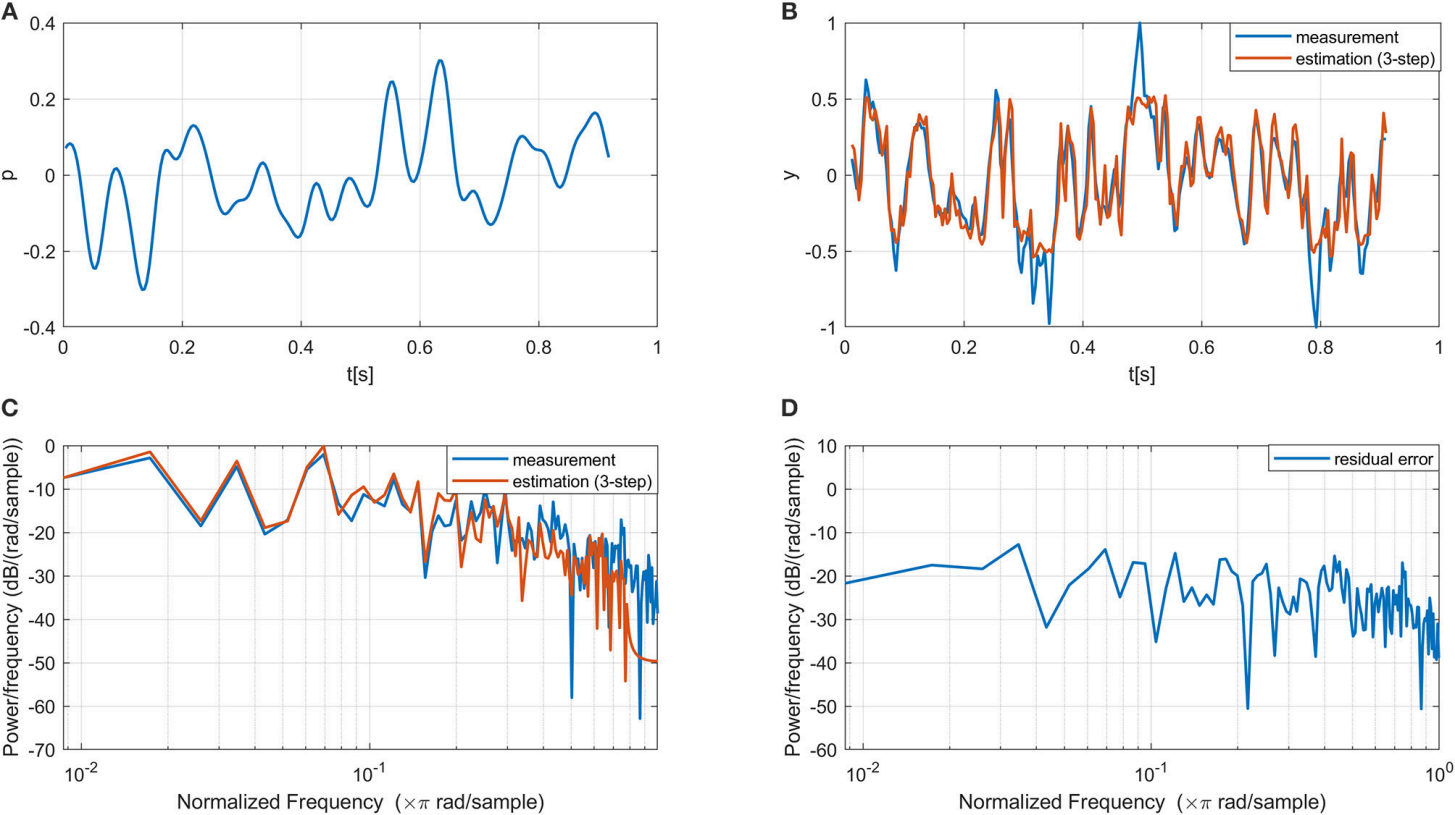
Example of the input, measured output, estimated output, and residual error signals using our proposed NARMAX-HNN model. **(A)** A period of the input signal, **(B)** Comparison between estimated and measured output time series, **(C)** Comparison between estimated and measured output power spectrum density **(D)** Power spectrum density of the residual error.

In line with the previous modeling study on the same datasets (Vlaar et al., 2018), only one ICA component with the highest SNR was selected as the output of the nervous system for each dataset for modeling. All selected components have their sources located in the primary sensorimotor areas in the contralateral hemisphere (i.e., left hemisphere as all participants are right-hand dominant), indicating that the ICA results are neurophysiological plausible. Figure 3 shows one period of the input and measured output signals from a typical subject. The estimated output signal from the multi-step (i.e., three-step) ahead prediction using the proposed NARMAX-HNN model is provided for comparison. The NARMAX-HNN captures the system's behavior well showing a similar waveform of the estimated output signal as the measured one. The residual error mainly appears in the high-frequency components, which is likely related to the fast dynamics of EEG oscillation as background noise in the system.

The model performances of NARMAX-HNN are summarized in the Table 1 for all tested subjects. The results are compared with those obtained by using the polynomial NARMAX and Volterra methods. Both the proposed NARMAX-HNN method (VAF: 92.33% ± 1.57%) and polynomial NARMAX method (VAF: 93.91% ± 1.54%) generated significantly better results (*P* < 0.0001 for both NARMAX-HNN vs. Volterra and polynomial NARMAX vs. Volterra) than the Volterra method for the one-step ahead prediction (VAF: 42.84% ± 13.78%). Although the one-step prediction results are comparable for the NARMAX-HNN and the polynomial NARMAX, the proposed NARMAX-HNN (VAF: 69.35% ± 11.90%) method yielded a significant better (*P* = 0.0015) long-term prediction (three-step MSA) than the polynomial NARMAX method (VAF: 47.09% ± 13.28%). Furthermore, the NARMAX-HNN contains 19 parameters, which is less than the polynomial NARMAX (25 parameters) and Volterra model (46 parameters). Finally, the numbers of parameters, associated with the number of model terms in the polynomial NARMAX and Volterra model, are optimal numbers that were determined by using an orthogonal forward regression (OFR) algorithm (Chen et al., 1989).

**Table 1:** Comparison of model performances, evaluated by variance accounted for in percentage (%), for the proposed NARMAX-HNN model, polynomial NARMAX (NARMAX-NP), and Volterra models.

**Participants**	**NARMAX-HNN (OSA)**	**NARMAX-HNN (MSA, 3-step)**	**NARMAX-NP (OSA)**	**NARMAX-NP (MSA, 3-step)**	**Volterra**
P1	94.37	63.44	95.52	57.08	38.37
P2	92.83	56.85	94.74	39.53	29.12
P3	90.95	67.16	92.95	31.17	32.18
P4	91.02	74.89	91.94	32.26	28.10
P5	92.58	82.31	94.04	61.57	53.74
P6	93.76	75.55	93.72	49.18	61.07
P7	93.08	74.32	95.73	65.35	54.30
P8	90.23	43.40	91.90	32.57	39.95
P9	90.36	77.16	92.24	37.98	26.35
P10	94.15	78.44	96.28	64.21	65.19
Mean	92.33	69.35	93.91	47.09	42.84
Std.	1.57	11.90	1.54	13.28	13.78

## Discussion and Conclusion

This work proposes an advanced modeling approach based on the NARMAX framework and a hierarchical neural network to model cortical activity in response to flexion and extension stretch perturbations at the wrist. As the human nervous system is comprised of multiple neuronal circuits resulting in complicated closed-loop non-linear behaviors in response to an external input, a realistic model should contain autoregressive variables to consider this closed-loop behavior. The NARMAX framework contains autoregressive variables as well as the interaction between the feedback and the input, which better reflects the non-linear closed-loop behavior of the nervous system than the Volterra model. As we demonstrated, both the proposed method, i.e., NAMRAX-HNN, and polynomial NARMAX method generated significantly better results than the Volterra model.

As shown in Table 1, the long-memory effect of autoregressive model increases the accumulative error of the long-term prediction, which results in a drop in VAF for multi-step ahead prediction using NARMAX. It has to be pointed out that multi-step ahead prediction is still a recognized challenge in the field of time series forecasting (Hussein et al., 2016), especially for cortical activity due to its fast dynamics (as shown in Figure 3) and a poor signal-to-noise ratio (Breakspear, 2017). Different from the commonly used polynomial NARMAX method, the proposed approach replaced the polynomial non-linear terms with a hierarchical neural network. Beyond the classical non-linear system identification approaches based on artificial neural network (Nelles, 2013), the hierarchical neural network here is built based on known neuroanatomical connections and corresponding transmission delays in neural pathways [i.e., the dorsal columns, (Carpenter and Sutin, 1983)]. This biologically inspired innovation significantly improves the long-term prediction of NARMAX modeling, showing better performance than the polynomial NARMAX in the estimation of 12 ms ahead EEG oscillation. Furthermore, our results are also better than the previous modeling study on the same datasets using the Volterra model (Vlaar et al., 2018) as demonstrated in Table 1. Finally, our proposed method has lower model complexity with a smaller number of parameters than the polynomial NARMAX and Volterra models. Although one may improve the model prediction ability by using an advanced machine learning approaches, such as deep learning, the purely mathematical or data-driven approaches are not able to generate a biologically realistic model with a reduced number of parameters.

In conclusion, the proposed method provides a novel solution to modeling of neural responses in the human nervous system with greater precision than polynomial NARMAX and Volterra models, in particular for long-term predictions. The proposed method considers both neuroanatomical pathways and physiological properties of the human nervous system. Therefore, it allows for the generation of neuroanatomically realistic models, which goes beyond purely mathematical or data-driven approaches. This study therefore breaks new ground in neurobiological system identification and modeling. In the future, we will apply this method to investigate changes in sensory and motor pathways following a unilateral brain injury such as hemiparetic stroke or hemiparetic cerebral palsy.

## Author Contributions

RT performed the data analysis and modeling under the supervision of YY and JD. YY drafted the manuscript with the assistance of RT. YY, FvdH, and JD contributed in problem identification. FvdH provided the experimental data. YY and JD participated in editing the manuscript.

### Conflict of Interest Statement

The authors declare that the research was conducted in the absence of any commercial or financial relationships that could be construed as a potential conflict of interest.
